# Vulnerability of Pampean Coastal Lizards to Global Change: Divergent Responses of Endemic Specialists and Widespread Generalists

**DOI:** 10.3390/biology15141152

**Published:** 2026-07-15

**Authors:** Juan E. Dajil, Carolina Block, Laura E. Vega, Pedro A. Garzo, Oscar A. Stellatelli

**Affiliations:** 1 Grupo Vertebrados, Instituto de Investigaciones Marinas y Costeras (IIMyC), Facultad de Ciencias Exactas y Naturales, Universidad Nacional de Mar del Plata—Consejo Nacional de Investigaciones Científicas y Técnicas, Mar del Plata B7602AYJ, Buenos Aires, Argentina; juanestebandajil@gmail.com (J.E.D.); cblock@mdp.edu.ar (C.B.);; 2 Grupo Geología de Costas y Paleoecología, Instituto de Investigaciones Marinas y Costeras, Facultad de Ciencias Exactas y Naturales, Universidad Nacional de Mar del Plata—Consejo Nacional de Investigaciones Científicas y Técnicas, Mar del Plata B7602AYJ, Buenos Aires, Argentina; pgarzo@agro.uba.ar; 3 Instituto de Geología de Costas y del Cuaternario “Dr. Enrique J. Schnack” (IGCC), Facultad de Ciencias Exactas y Naturales, Universidad Nacional de Mar del Plata—Comisión de Investigaciones Científicas, Mar del Plata B7602AYJ, Buenos Aires, Argentina

**Keywords:** endemic reptile, global warming, landscape transformation, *Liolaemus*, Pampas

## Abstract

The 21st century is defined by converging anthropogenic and biophysical stressors. This study assessed the vulnerability of the endemic specialist lizard *Liolaemus multimaculatus* and the habitat generalist *L. wiegmannii* to climate change and land-use/land-cover (LULC) transformation within the Pampean Eastern Dune Barrier up to 2050. Using satellite data, LULC spatial projections, and ecological niche models (ENMs), we quantified habitat dynamics and projected future climatic suitability. Historical analysis (1994–2022) revealed a 20% retraction of active dunes driven by exotic afforestation and urban growth. Projections for 2050 indicate an intensification of these trends, with urban areas accounting for nearly 26% of the regional territory, leading to an additional 17% loss of active dunes. Abundance modeling predicted a decline in *L. multimaculatus* within the remaining active dunes, while ENMs projected a near-total contraction of climatically suitable areas. Crucially, these correlative models may overlook potential physiological or behavioral adjustments; however, the limited dispersal capacity and physical landscape barriers inherent to this specialist likely preclude effective niche tracking. These findings confirm that ecological specialization heightens sensitivity to global change, creating a “double threat” for endemic species. Protecting remnant active dune patches is essential to mitigate the projected collapse of these range-restricted lineages.

## Simple Summary

Coastal dunes in the Argentine Pampas face growing pressure from human activities and climate change. This study explores how human-driven changes and climate shifts impact two native lizard species in these coastal dunes. We compared the sand-dwelling specialist lizard, *Liolaemus multimaculatus*, with the more versatile *Liolaemus wiegmannii*. By analyzing satellite maps and future climate patterns through 2050, we found that invasive trees and expanding cities have already eliminated 20% of the lizards’ natural dunes. Our results suggest that by 2050, urban growth will claim another 17% of this habitat. While our abundance models project a reduction within remaining active dune patches, our climate niche models suggest a near-total loss of climatically suitable areas for the specialist. Importantly, these correlative projections do not account for physiological acclimatization, behavioral plasticity, or microhabitat buffering, which may modulate vulnerability. Conversely, the generalist lizard may survive by moving inland toward the Arid Diagonal. These findings demonstrate that species with strict habitat needs are at extreme risk. To prevent the extinction of these native species, it is vital to protect the remaining wild coastal dunes. This research provides a framework for understanding which animals are most vulnerable to our changing world, thereby facilitating better conservation planning.

## 1. Introduction

The 21st century is characterized by the convergence of anthropogenic and biophysical stressors collectively termed “global change”, which integrates climate change and land-use/land-cover (LULC) shifts [1,2,3]. At a global scale, these processes represent the primary drivers of biodiversity loss and mass extinctions [4,5,6]. Although these factors have historically been studied in isolation, they interact synergistically because habitat degradation reduces the capacity of species to redistribute in response to advancing climatic shifts [7,8,9]. In this context, endemic and range-restricted species face a disproportionately high extinction risk due to limited dispersal capabilities and an inability to track suitable climatic niches [10,11]. Such isolation is exacerbated by LULC changes that promote fragmentation, directly reducing functional connectivity and preventing populations from reaching thermal refugia [12,13,14,15].

The magnitude of global change impacts often depends on the degree of habitat specialization; specialist species exhibit heightened vulnerability compared to generalists, which frequently persist in modified landscapes [16,17]. Global projections for 2050 suggest that changes in natural LULC and urban expansion will remain the primary drivers of terrestrial vertebrate declines [18,19,20]. These trends may be obscured by a “delayed extinction debt,” where current land-cover changes commit populations to future collapse before physical habitat loss is complete [21]. Furthermore, intensified warming may decouple ecological interactions, leading to co-extinction cascades [22]. Ultimately, these pressures are likely to result in faunal homogenization, dominated by a few generalist species capable of persisting in human-modified environments [17,23].

For reptiles, global diversity is projected to undergo significant spatial reconfigurations by 2050, with species richness declining in tropical and subtropical lowlands due to thermal stress exceeding critical physiological limits [24]. As ectotherms, squamate populations are highly vulnerable to climate change; their persistence and viability are directly contingent upon thermal and hygric habitat conditions that are currently being altered by global warming [12,25,26]. The combination of physiological sensitivity and low mobility creates a “double threat” that accelerates population collapse in altered ecosystems [6,27,28,29]. While some models anticipate poleward or upslope range expansions for generalist taxa, these gains are often offset by the loss of specialized endemic lineages in isolated systems [30]. It is estimated that up to 20% of reptile species could face global extinction by mid-century [25].

In the Southern Cone of South America, these challenges are equally critical. In Mediterranean ecosystems, such as the Chilean matorral, drastic reductions in reptile diversity are projected under high-emission scenarios [31]. Within Argentina, vulnerability is heterogeneous; while climate alters suitable areas, LULC change reduces the actual probability of persistence [32,33]. A system particularly affected is the Pampean coastal dune barrier, which has been historically degraded by two major LULC changes: urbanization and dune fixation through exotic afforestation [34,35,36,37]. In this ecosystem, structural landscape modifications and thermal alterations differentially impact sympatric lizards; for instance, the introduction of exotic species alters thermoregulatory efficiency in a species-specific manner [38,39]. Future climate modeling of nine endemic and threatened species in the Pampas indicates that under pessimistic greenhouse gas emission scenarios (RCP 8.5), most taxa are projected to lose over 60% of their suitable habitat by 2080, significantly elevating their risk of extinction [6,40].

The Sand Dune Lizard, *Liolaemus multimaculatus*, is a specialist endemic categorized as “Endangered” by the IUCN [41]. Its survival depends critically on extensive patches of active dunes, showing high sensitivity to habitat loss [42,43]. In contrast, *Liolaemus wiegmannii* is a habitat generalist capable of utilizing human-modified environments, although its thermoregulatory efficiency may still be compromised in structurally altered habitats [34,36,38,44]. Despite the urgency, a critical knowledge gap persists regarding the future impacts of climate change on Argentine Pampean reptiles, and data are virtually non-existent for future projections involving LULC changes [40]. Understanding these interactions is imperative for designing conservation strategies that ensure habitat connectivity in the Anthropocene [45,46]. However, it is important to emphasize that while projections derived from both LULC and climatic modeling frameworks are inherently correlative [47], these tools serve as essential proxies for evaluating species risk. Consequently, while these models may not fully capture critical biological responses—such as physiological acclimatization potential, behavioral plasticity, or the microhabitat buffering capacity that species might employ to mitigate environmental stress [48]—they provide the necessary baseline to address the aforementioned knowledge gaps.

The main objective of this study was to assess the vulnerability of *L. multimaculatus* and *L. wiegmannii* to land-use and land-cover changes and climate change through the mid-21st century. Specifically, we aimed to: (1) quantify the spatiotemporal dynamics of the sand dune landscape (1994–2050); (2) model the current and future abundance distribution of both species based on land use and land cover patterns; and (3) project current and future climatic suitability using ecological niche models (ENMs). We hypothesized that (H1) ecological specialization determines differential sensitivity to LULC changes, predicting that (P1) the impacts of landscape change will be significantly higher for *L. multimaculatus* and (P2) its distribution will decline drastically compared to that of the generalist *L. wiegmannii*. Furthermore, we hypothesized that (H2) climate change impacts are more severe in reptiles with restricted ranges and specific thermal requirements, leading to the prediction that (P3) ENMs will project a niche contraction for *L. multimaculatus* and (P4) the proportion of high-risk areas will be higher for this specialist species when comparing both global change drivers.

## 2. Materials and Methods

### 2.1. Study Area

**Figure 1:**
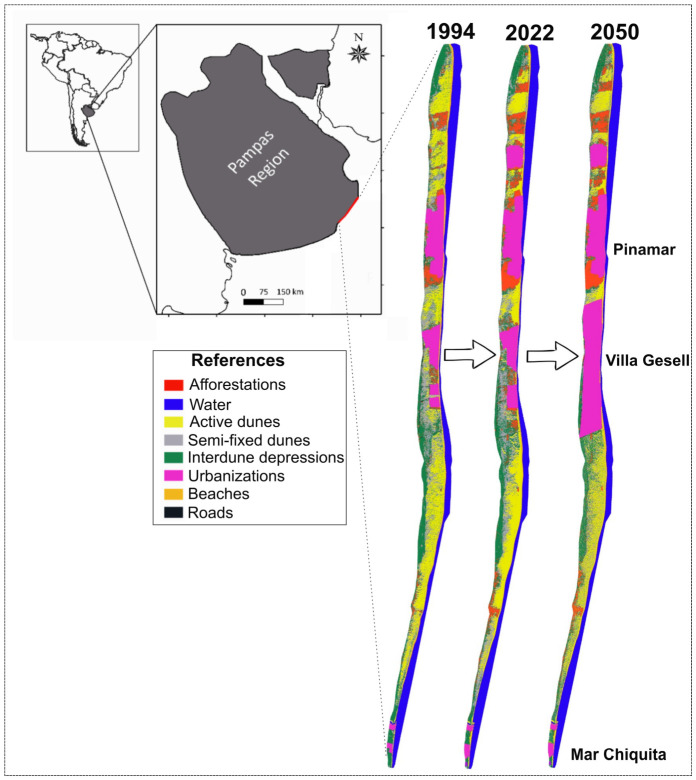
Study area and spatiotemporal dynamics of the Eastern Dune Barrier, Argentina. The left panel shows the Pampas Region (gray) within South America and the location of the coastal study area (red line). Right panels display thematic land-use/land-cover (LULC) maps for 1994, 2022, and a projected scenario for 2050. These maps illustrate the LULC changes highlighting the expansion of urbanization (pink) and afforested dunes (red) at the expense of active dunes (yellow).

The study was conducted within the temperate coastal Pampa dune landscape of Buenos Aires province, Argentina, focusing specifically on the Eastern Dune Barrier. The study area encompasses a total of 46,203 hectares (ha), which extends for approximately 192 linear km from San Clemente del Tuyú (36°18′ S; 56°45′ W) to La Caleta (37°44′ S; 57°27′ W; datum: WGS84) [36,49]. Sampling sites were established across a longitudinal extent of approximately 130 km from Punta Médanos (36°53.00′30.96″ S, 56°40.00′50.90″ W) in the north to La Caleta (37°47.00′26.42″ S, 57°27.00′40.90″ W) in the south (Figure 1). This area encompasses a representative portion of this dune system that includes the main human settlements such as Pinamar, Villa Gesell and Mar Chiquita village and natural areas that are included in reserves such as the Parque Atlántico Mar Chiquito UNESCO–MAB Biosphere Reserve and the Faro Querandí Nature Reserve. The dune system exhibits a typical geomorphological zoning pattern from sea to upland, encompassing the upper beach, active fore-dunes, active inland dunes, semi-fixed dunes, and interdune depressions [50]. This region experiences a temperate oceanic climate, characterized by humid weather, an average maximum summer temperature of 33 °C, an average winter temperature of 9 °C, and mean annual precipitation ranging from 800 to 1000 mm [51,52,53]. It is noteworthy that these coastal dunes represent one of the last remnants of native Pampean grasslands in dune environments, though significant structural alteration, including the replacement of native plants with rapidly growing exotic trees for dune fixation associated with human settlements, has impacted the ecosystem [54,55].

### 2.2. Satellite Image Processing and LULC Classification

A Geographic Information System (GIS) was developed using the open-source software QGIS (version 3.14.0-Pi) to characterize the coastal dune landscape. Past and current LULC identification was performed via supervised classification within two OLI-TIRS Landsat 8 images (30 m pixel resolution) acquired in 1994 and 2022, respectively (Path: 223; Row: 086; UTM/WGS-84; UTM zone 21S; available from USGS Earth Explorer; https://earthexplorer.usgs.gov/ (accessed on 13 September 2023)). According to Garzo et al. [37], intra-annual variability in satellite time series for the study area does not exhibit statistically significant differences among mean seasonal spectral values. Thus, intra-annual variability may be considered negligible, whereas inter-pixel variability is more representative, tending to reflect smoothly progressive change patterns beyond seasonal fluctuations. The Semi-Automatic Classification Plugin (SCP), an open-source QGIS tool [56], was used to preprocess the imagery, extract spectral signatures, and execute the supervised classification workflow. Classification was performed using the Spectral Angle Mapper (SAM) algorithm, which was selected for its proficiency in discriminating between spectrally similar classes [57]. Urban areas, roads, and beaches were digitized from the Instituto Geográfico Nacional (IGN) vector database (https://www.ign.gob.ar/, accessed on 19 July 2022) and manually rectified using Google Earth imagery, then rasterized and added to the final maps. The resulting thematic maps of 1994 and 2022 (Figure 1) delineated eight LULC categories: (1) Active Dunes (sparsely vegetated upper beach, active foredunes, and active inland dunes); (2) Semi-Fixed Dunes (continuous natural grassland, clump herbs, and shrubs); (3) Interdune Depressions (flood-associated vegetation); (4) Afforested Dunes (exotic trees); (5) Urban Areas; (6) Beaches (intertidal zone, foreshore and lower backshore); (7) Water Bodies; and (8) Roads [34,43,44]. Classification accuracy was evaluated via error matrix analysis and the Kappa index [58]. The assessment yielded an overall accuracy of 76.76% (kappa = 0.73) for the historic (1994 map) and a significantly higher accuracy of 91.71% (kappa = 0.88) for the current (2022) thematic map. We utilized the Kappa index to evaluate classification accuracy, following common remote sensing practices. However, it is important to note that the field of LULC analysis has increasingly transitioned toward more informative accuracy measures, such as quantity and allocation disagreement. While Kappa remains a standard output of the MOLUSCE (version 5.0.0) classification workflow, we recognize its inherent limitations and acknowledge that future accuracy assessments should prioritize disagreement-based metrics to provide a more comprehensive interpretation of classification error [59].

### 2.3. Spatiotemporal Dynamics of LULC Scenarios

LULC projections up to 2050 were developed based on the thematic maps obtained in Section 2.2. These projections provide the spatial distribution of five land-cover categories: (1) Active Dunes; (2) Semi-Fixed Dunes; (3) Interdune Depressions; (4) Afforested Dunes; and (5) Urban Areas. Based on the thematic maps of the spatial distribution of LULC classes (for 1994 and 2022), the MOLUSCE (Module for Land Use Change Evaluation) plugin for QGIS [60] was used to analyze 28-year intervals at a spatial resolution of 30 m. To account for pixel-scale transition potential, five raster layers were incorporated as explanatory variables: a Digital Elevation Model (DEM, 30 m/pixel, sourced from USGS), DEM-derived slope and aspect maps, a distance-to-urban-centers map, and the Normalized Difference Vegetation Index (NDVI) calculated from 2022 Landsat 8 OLI/TIRS imagery [61]. The absence of multicollinearity among variables was verified using Pearson’s correlation coefficient (r < 0.6; Supplementary Table S1). Given the high computational demand and to optimize model learning efficiency, the study area was transversally subdivided into four independent analysis zones of similar extent. Transition potential maps were modeled using Artificial Neural Networks (ANN), trained with the following hyperparameters: learning rate = 0.05, iterations = 1000, hidden layers = 8–4, and momentum = 0.01. Based on these parameters, the transition-potential map for the 1994–2022 period was modeled and validated against the 2022 thematic map. Model performance was assessed using the Kappa index, yielding values of 0.84 for Zone 1, 0.88 for Zone 2, 0.85 for Zone 3, and 0.90 for Zone 4. These results are consistent with values reported in previous studies applying the same methodology in other systems [37,62]. This validation enabled the generation of a new 28-year analysis period, projecting land-cover transitions for 2022–2050. The 2050 urbanization projection was based on governmental land-use planning criteria. Specifically, for the Villa Gesell district—a representative urban center of the eastern barrier dunes (Figure 1)—we incorporated the expansion projected in the Municipal Land-Use Plan [37,63,64]. For the remaining districts (Mar Chiquita, Pinamar, and La Costa), current regulatory restrictions (Provincial Decree-Law No. 8912/1977 [65] and Decree No. 3202/2006 [66]) were applied, prohibiting urban expansion over natural dunes, as none meet the minimum requirement of a 5 km coastal dune reserve frontage established by governmental regulations. Subsequently, the maps for the four zones were integrated into a single projected map for 2050, incorporating static layers and projected urbanization. The three LULC maps (1994, 2022, and 2050; Figure 1) were analyzed in MOLUSCE to obtain land-cover change statistics for the 1994–2022 and 2022–2050 periods. The 2050 LULC projection implemented here represents a single deterministic trajectory derived from historical transition rates (1994–2022) using a Markov chain and artificial neural network approach in MOLUSCE. Consequently, this approach does not allow for the parameterization of alternative socioeconomic scenarios—such as conservative versus intensive urban growth—and the uncertainty associated with future socioeconomic volatility is not formally propagated into our vulnerability estimates.

### 2.4. Spatiotemporal Dynamics of Lizard Abundances

To estimate changes in lizard abundance, we projected the distribution of both *Liolaemus* species at each timestep of the scenarios. The abundance dataset was obtained from Dajil et al. [43], and is available on Zenodo (https://doi.org/10.5281/zenodo.21348143) [67]. The spatial distribution of abundance for both species was projected for 1994, 2022, and 2050 by extrapolating predictive models based on the spatial scales and landscape variables previously identified as significant for *L. multimaculatus* and *L. wiegmannii* [see 43]. Specifically, for *L. multimaculatus*, we employed a model based on landscape variables calculated using windows (landscape units) with a 500 m radius. The variables included the total area of active dunes (AD), total area of semi-fixed dunes (SD), total area of afforested dunes (FD), and (4) total area of beach (BCH). For *L. wiegmannii*, the model utilized variables obtained from 100 m radius windows, considering the geometric complexity of semi-fixed dune patches (SSD) and the number of semi-fixed dune patches (NPS). Explanatory variables for each timestep were obtained using circular moving windows in FRAGSTATS (version 4.2.681) [68], generating continuous raster layers for each landscape metric across the entire study area. We applied the Generalized Linear Models (GLMs) described by Dajil et al. [43] using the glmmTMB package (version 1.1.7) [69] in R (version 4.3.1) [70]. These models were adjusted by excluding random effects and restricting predictions to the specific habitats of each species: active dunes for *L. multimaculatus* [42] and semi-fixed dunes for *L. wiegmannii* [34]. Random effect terms (spatial hierarchical nesting, temporal autocorrelation, and zero-inflation) reflect properties of the sampling design and therefore cannot be projected onto unsampled raster cells. Consequently, only the fixed effect component of the models, capturing the relationship between landscape variables and relative abundance, was retained for spatial extrapolation. Validation metrics reported in the Section 3 correspond to the full models, including random effects, as described in Dajil et al. [43]. To account for the potential spatial dependence inherent in data collected across a 130 km coastal strip, our abundance models explicitly incorporated the spatial structure of the sampling design through a nested hierarchical structure, with transects nested within spatial sampling units. This approach accounts for the expected lack of independence between observations within the same spatial units. Furthermore, we evaluated the model fit and the presence of residual spatial autocorrelation using the DHARMa package (version 0.4.6). We inspected the distribution of simulated residuals via quantile–quantile (Q-Q) plots, examined the relationship between residuals and predicted values for systematic patterns, and performed non-parametric dispersion tests. The spatial predictive performance of the models was evaluated through block cross-validation using a leave-one-transect-out scheme [71]. Pearson’s correlation, pseudo-R^2^, RMSE, and MAE were calculated between observed and predicted values. Subsequently, the landscape metric raster layers were imported using the terra package (version 1.8.29) [72], and spatially explicit predictions were performed using the predict() function from the raster package (version 3.6.26) [73], generating projected abundance maps for each species and analyzed year.

### 2.5. Ecological Niche Modeling and Climate Change Impact

Environmental data. Current and future environmental variables were sourced from WorldClim version 2.1 at the highest available resolution of 30 arc-seconds (~1 km^2^). The dataset comprised 19 bioclimatic variables derived from temperature and precipitation, alongside the latitude variable [74]. For *L. multimaculatus*, the following variables were selected: annual mean temperature (BIO1), minimum temperature of the coldest month (BIO6), temperature annual range (BIO7), and annual precipitation (BIO12). For *L. wiegmannii*, the selection included annual mean temperature (BIO1), temperature seasonality (BIO4), annual precipitation (BIO12), precipitation seasonality (BIO15), and precipitation of the warmest quarter (BIO18). Variable selection was based on ecological relevance for reptiles and correlation analyses to avoid multicollinearity (Pearson’s *r* < 0.6; Supplementary Table S2). The final number of retained bioclimatic variables differed between species as a consequence of differences in sample size and geographic range. *Liolaemus wiegmannii* showed a larger number of occurrence records and a broader geographic distribution than *L. multimaculatus*, allowing one additional parameter to be incorporated without risking overfitting. In contrast, given the smaller number of occurrences and more restricted distribution of *L. multimaculatus*, a reduced variable set was used to maintain an adequate ratio between the number of parameters and observations [75]. Regarding the biological relevance of the selected variables, our results are consistent with recent studies identifying temperature seasonality and precipitation patterns as primary drivers of suitable habitat distribution for Pampean reptiles [40].

Niche modeling. To project climate suitability under climate change scenarios, occurrence records were compiled across the entire distribution range of both species from literature sources. For *L. multimaculatus*, records were obtained from Cei [76], Vega and Bellagamba [77,78], Etheridge [79], Stellatelli et al. [80], Abdala et al. [81], and Block et al. [42] (Supplementary Figure S1). For *L. wiegmannii*, data were sourced from Cei [76], Achaval and Olmos [82], Vega and Bellagamba [77], Martori et al. [83], Etheridge [79], Scrocchi et al. [84], Stellatelli et al. [38,39], Block et al. [34], Verrastro et al. [85], Villamil et al. [86], Abdala et al. [81], and Williams et al. [87] (Supplementary Figure S2). Historical records (2008–2024) from the Vertebrate Research Group (IIMyC, FCEyN, UNMdP-CONICET) and the Vertebrate Group Herpetological Collection database were also included [88]. Additionally, community science platform records were incorporated for *L. wiegmannii* from GBIF [89], iDigBio [90], and iNaturalist [91], retrieved programmatically through their respective APIs using the rgbif (version 3.8.3) [92], ridigbio (version 0.4.1) [93], and rinat (version 0.1.10) [94] packages in R (version 4.3.1) [70]. Records were obtained via direct API queries rather than through registered downloads, and therefore no GBIF download DOI is associated with this dataset. The complete set of retrieved records is available in the Zenodo repository associated with this study [67]. 

Prior to the calibration of the potential distribution models, all occurrence records were subjected to an exhaustive taxonomic, spatial, and temporal cleaning process using the bdc (version 1.1.5) [95] and CoordinateCleaner (version 3.0.1) [96] packages in R (version 4.3.1) [70]. We removed duplicate records, coordinates with missing, erroneous, or low-precision data, records located outside the expected geographical range or in marine environments, as well as points associated with museums, biological institutions, and administrative centroids. Furthermore, taxonomic consistency was verified for all scientific names, and the geographical distribution of both species was manually reviewed to exclude any dubious records. This procedure generated a refined, high-quality occurrence database suitable for robust model calibration. To account for potential spatial autocorrelation (SAC)—which can lead to overoptimistic performance estimates in spatial models—we employed spatial block cross-validation with 5 folds. This procedure partitions the data based on geographic proximity, ensuring spatial independence between the training and testing sets and providing a rigorous evaluation of the model’s predictive performance. After data cleaning (removal of duplicates and erroneous coordinates), 282 records remained for *L. multimaculatus* and 703 for *L. wiegmannii*.

The calibration area (accessible area “M”) was defined as a Minimum Convex Polygon (MCP) encompassing all occurrence records for each species, to which a species-specific buffer was added to represent the area historically accessible via dispersal, following the conceptual framework of Barve et al. [97]. For the habitat specialist *L. multimaculatus*, which exhibits restricted dispersal capacity across non-suitable matrices and is highly sensitive to habitat loss, a 50 km buffer was applied to restrict the calibration area to the temperate coastal dune system where the species maintains historical and contemporary connectivity [42,43]. Conversely, a 100 km buffer was applied for *L. wiegmannii*, a habitat generalist with a broader distribution across coastal pampas and inland ecoregions of Argentina [86]. This larger buffer for *L. wiegmannii* accounts for its greater vagility and ability to capitalize on landscape heterogeneity, as supported by its presence across more diverse and fragmented coastal habitats [34,43]. These buffer distances were selected to approximate the historically accessible area for each species while avoiding the inclusion of regions effectively isolated by historical or ecological barriers—such as unsuitable inland matrices or extensive anthropogenic fragmentation—that could introduce spurious sampling bias in model calibration.

Models were fitted using three algorithms via the flexsdm R package (version 1.3.8) [98]: Random Forest (RAF), Maximum Entropy (MaxEnt), and Generalized Linear Models (GLM). To account for potential spatial autocorrelation (SAC), which can lead to overoptimistic performance estimates in spatial models [71], we performed all model calibrations using spatial block cross-validation (part_sblock) with 5 folds. This procedure partitions the data based on geographic proximity, ensuring spatial independence between the training and testing sets and providing a rigorous evaluation of the model’s predictive performance under the assumption of spatial stationarity. Model performance was evaluated using AUC, TSS, the Jaccard index, the Boyce index, and IMAE; the resulting metrics for all algorithm–species combinations are detailed in Supplementary Table S3. Although MaxEnt yielded slightly higher absolute values for some metrics in initial runs, we selected Random Forest as the primary algorithm for both species. This decision was based on the numerical stability and robustness of Random Forest throughout the entire analytical pipeline—including threshold sensitivity analyses and ensemble projections—whereas MaxEnt exhibited signs of model degeneration (i.e., failure to identify unique environmental thresholds) in the restricted calibration area of *L. multimaculatus*. Continuous suitability surfaces were converted into binary presence/absence maps using the threshold that maximizes the sum of sensitivity and specificity (max_sens_spec; mean threshold = 0.318 ± 0.243 for *L. multimaculatus* and 0.151 ± 0.077 for *L. wiegmannii*, across spatial cross-validation partitions). To evaluate the robustness of area-based conservation conclusions to threshold choice, we additionally computed four alternative threshold criteria (equal sensitivity-specificity, maximum Jaccard index, maximum Sorensen index, and lowest presence threshold) for both species; results of this sensitivity analysis are reported in Supplementary Table S4.

Future climate projections were based on an ensemble of four General Circulation Models (GCMs) from the CMIP6 archive (https://worldclim.org/data/cmip6/cmip6climate.html, accessed on 3 July 2026): MPI-ESM1-2-HR, ACCESS-CM2, IPSL-CM6A-LR, and EC-Earth3-Veg. The selection of MPI-ESM1-2-HR was not arbitrary: this model combines higher spatial resolution with improved biases in upper-level winds, jet stream positioning, and precipitation patterns relative to its lower-resolution counterpart, making it well-suited for impact studies in coastal landscapes with environmental heterogeneity [99]. Regional evaluations of CMIP6 models in South America have shown that MPI-ESM1-2-HR ranked among the best-performing models for precipitation in southeastern South America [100], a region encompassing our study area, while ACCESS-CM2 and IPSL-CM6A-LR have been identified as suitable for assessing regional climate change impacts across South America [101], and EC-Earth3-Veg has similarly shown good regional performance in South America [100]. We acknowledge that reliance on a single GCM does not capture inter-model uncertainty, which can be as large as, or larger than, that introduced by emission scenarios [102,103]. For each species, GCM, and Shared Socioeconomic Pathway (SSP) scenario (SSP2-4.5 and SSP5-8.5), future bioclimatic layers were restricted to the calibration area previously defined for current conditions. Future suitability was summarized as the ensemble mean across the four GCMs, and inter-GCM uncertainty was quantified as the standard deviation across models at each grid cell. All analyses were conducted in R (version 4.3.1) [70], and maps were visualized using QGIS (version 3.14.0-Pi).

## 3. Results

### 3.1. Spatiotemporal Dynamics of LULC Scenarios

Analysis of historical, current, and projected LULC patterns revealed a significant and continuous loss of native habitats, specifically active and semi-fixed dunes, across both study periods (1994–2022 and 2022–2050). During the historical interval (1994–2022), afforested dunes expanded by 95% (2184 ha), increasing their total landscape share from 4.95% to 9.68% (Table 1; Figure 1). Concurrently, urban areas grew by 28% (1563 ha), rising from 12.18% to 15.56% of the study area. In contrast, active dunes underwent a 20% retraction (a loss of 2123 ha), while semi-fixed dunes declined by 10% (739 ha). These natural covers dropped from 22.58% to 17.98% and 15.91% to 14.31%, respectively (Table 1; Figure 1). For the projected period (2022–2050), trends indicate an intensification of native habitat loss and urban expansion, though exotic afforestation is expected to stabilize or slightly decline (Table 1; Figure 1). Projections suggest a further 17% decrease in active dune cover (1403 ha) and a 23% reduction in semi-fixed dunes (1499 ha), reaching 14.95% and 11.07% of the total surface area by 2050. Simultaneously, urban areas are projected to increase by 67% (4809 ha), ultimately accounting for 25.97% of the regional territory (Table 1; Figure 1).

**Figure 2:**
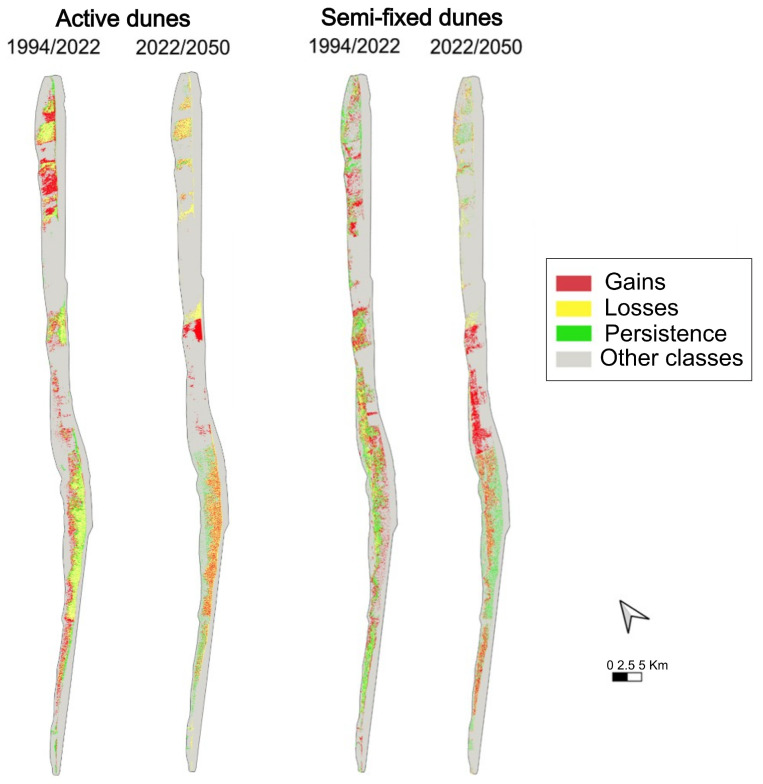
Spatiotemporal dynamics of dune cover classes in the Eastern Dune Barrier, Pampas Region, Argentina. Maps illustrate transitions for the historical (1994–2022) and projected (2022–2050) periods: The left panels show the spatial distribution of gains, losses, and persistence for active dunes; the right panels show the same for semi-fixed dunes. In both panels, green pixels indicate area gains (recruitment from other classes), red pixels represent area losses (conversion to other classes), and yellow pixels denote stable areas (persistence) for the respective category. Gray areas represent all other land-use/land-cover classes.

Analysis of historical and projected transitions reveals that during the 1994–2022 period, 79.6% (6150 ha) of the original active dune area remained stable (Figure 2). Concurrently, a gross loss of 4281 ha was recorded, primarily driven by transitions to semi-fixed dunes (52%) and interdune depressions (16%), while conversion to urban areas and exotic afforestation each accounted for 14% (Figure 2). Conversely, a gain of 2158 ha in active dunes by 2022 was attributed to recruitment from semi-fixed dunes (61%), interdune depressions (26%), and, to a lesser extent, beach-to-dune transitions (11%) (Figure 2). In the 2022–2050 scenario, 73.6% (5684 ha) of active dune cover is projected to persist (Figure 2). An estimated loss of 2625 ha is expected, linked to transitions toward semi-fixed dunes (61%) and urban expansion (27%), with minor contributions to interdune depressions (8.5%) and afforested dunes (3.4%) (Figure 2). Projected gains of 1222 ha would stem largely from semi-fixed dunes (78%), followed by interdune depressions (16%) (Figure 2). Although these transitions demonstrate that the landscape exhibits a bidirectional exchange between dune classes at a localized scale, the aggregated data reveal a clear, unidirectional trajectory toward a net negative balance for the active dune class.

Regarding semi-fixed dunes, 40.3% (2965 ha) of the original 1994 surface persisted through 2022 (Figure 2). The recorded loss of 4386 ha was driven by conversion to interdune depressions (41%), active dunes (30%), and exotic afforestation (22%), while urban areas and beaches represented less than 10% of this retraction (Figure 2). Simultaneously, a gain of 3647 ha was primarily explained by transitions from active dunes (61%) and interdune depressions (34%) (Figure 2). Under the 2022–2050 model, 44.5% (2942 ha) of the semi-fixed dune area is projected to persist (Figure 2). The projected loss of 3670 ha is associated mainly with urban expansion (49%), followed by transitions to active dunes (26%) and interdune depressions (22%) (Figure 2). Projected gains of 2172 ha would derive fundamentally from the stabilization of active dunes (73%) and transitions from interdune depressions (26%) (Figure 2). Collectively, while these historical and projected patterns reflect a gross turnover indicative of bidirectional exchange, the net outcome is a unidirectional reduction in semi-fixed dune cover, primarily driven by land-use pressures such as urban expansion and afforestation.

### 3.2. Spatiotemporal Dynamics of Lizard Abundance

**Figure 3:**
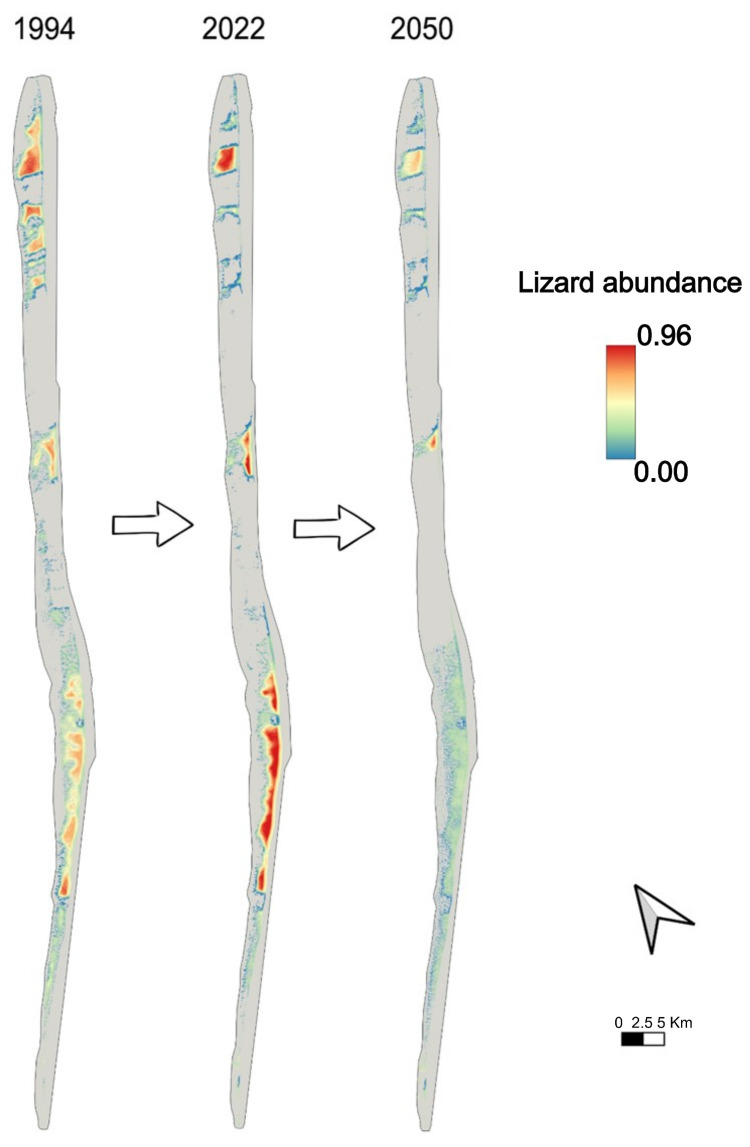
Predictive abundance maps for *Liolaemus multimaculatus* in the Eastern Dune Barrier, Pampas Region, Argentina. Maps illustrate model-predicted relative abundance for the years 1994, 2022, and 2050. White arrows signify the chronological sequence. The black and white arrow in the bottom right indicates orientation (North). The color ramp indicates relative abundance from low (blue, 0) to high (red, 0.96). Note that predictions are generated solely for active dune areas; gray regions denote all other land-use/land-cover classes that are unsuitable for this species.

Spatial modeling of relative abundance revealed distinct patterns for the two studied species. For *L. multimaculatus*, predictive maps demonstrated a strong dependency on LULC patterns (Figure 3). In 1994, the highest abundances were associated with extensive, continuous patches of active dunes in the northern sector of the study area. However, for the 2022 and 2050 scenarios, the models predicted a severe decline in these core areas, driven by the expansion of urban areas and exotic afforestations (Figure 3). Notably, even within protected areas such as the Faro Querandí Municipal Nature Reserve, a reduction in abundance is projected by 2050. This decline is a direct consequence of habitat loss via dune stabilization (i.e., the successional transition from active to semi-fixed dunes). Overall, the models indicate a gradual historical decline in *L. multimaculatus* abundance that is forecasted to intensify through 2050 due to the synergistic effects of urban sprawl and afforestation. Regarding model performance, spatial cross-validation (leave-one-transect-out) applied to the Zero-Inflated Poisson (ZIP) models for *L. multimaculatus* yielded global validation metrics (r = 0.21; pseudo-R^2^ = 0.04; RMSE = 0.50; MAE = 0.34 individuals per unit). While these metrics indicate that the models explain a low fraction of total variance, the results remain ecologically coherent. Despite the predictive power explaining less than 5% of the variation in independent data, the spatial outputs presented in Figure 3 effectively identified relative abundance gradients consistent with landscape-scale transitions. In contrast, the predicted abundance for *L. wiegmannii* remained relatively stable across space and time, showing a closer association with the availability of semi-fixed dunes. However, the model for *L. wiegmannii* exhibited marginal predictive power (r = 0.11; R^2^ ≈ 0.01; RMSE = 0.72; MAE = 0.54), suggesting that the evaluated landscape variables have limited explanatory capacity for this species compared to the dynamics observed for *L. multimaculatus*. Diagnostic evaluations of the model residuals did not indicate significant deviations from the model assumptions. The Q-Q plots of the simulated residuals showed no meaningful departures from the expected distribution, and the plots of residuals against predicted values exhibited no systematic patterns, indicating that the models adequately captured the underlying data structure (Supplementary Figures S3 and S4).

### 3.3. Ecological Niche Modeling and Climate Change Impacts

**Figure 4:**
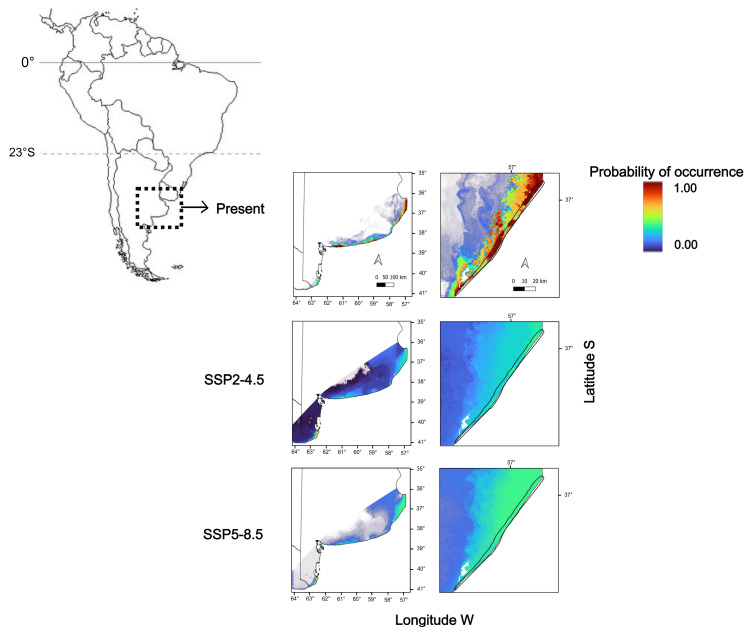
Ecological Niche Models (ENMs) for *Liolaemus multimaculatus*. The maps illustrate the predicted probability of occurrence for the species under current climatic conditions (**top row**), a moderate 2050 climate change scenario (SSP2-4.5; **middle row**), and a pessimistic 2050 scenario (SSP5-8.5; **bottom row**). The left column displays broad-scale geographic distribution predictions, and the right column provides high-resolution maps of the primary study area along the Eastern Coastal Dune Barrier of the Pampas region, Argentina. The black arrow on the locator map of South America points from the dashed box enclosing the study region to the current distribution map (“Present”). Stylized arrows within the map panels indicate North orientation. The color ramp represents the probability of occurrence, ranging from low (blue, 0.00) to high (red, 1.00).

**Figure 5:**
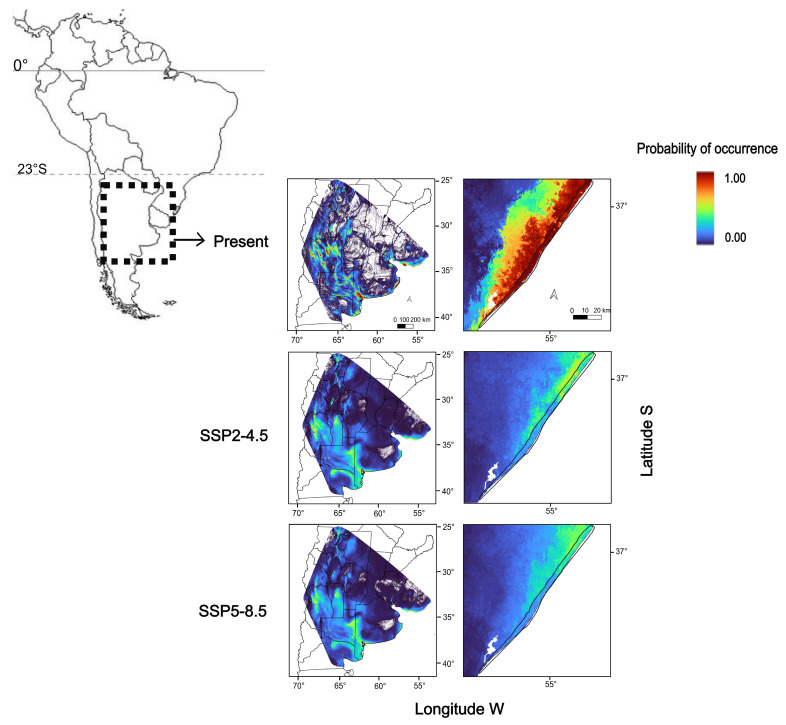
Ecological Niche Models (ENMs) for *Liolaemus wiegmannii*. The maps illustrate the predicted probability of occurrence for the species under current climatic conditions (**top row**), a moderate 2050 climate change scenario (SSP2-4.5; **middle row**), and a pessimistic 2050 scenario (SSP5-8.5; **bottom row**). The left column shows the broad-scale geographic distribution predictions across southern South America. The right column provides a high-resolution view of the study area within the Eastern coastal dune barrier of the Pampas region, Argentina. The black arrow on the locator map of South America points from the dashed box enclosing the study region to the current distribution map (“Present”). Stylized arrows within the map panels indicate North orientation. The color ramp represents the probability of occurrence, ranging from low (blue, 0.00) to high (red, 1.00).

Ecological Niche Models (ENMs) were employed to isolate the specific effects of climate change on the potential distribution of both study species (Figure 4 and Figure 5). The ecological niche models demonstrated high predictive performance across all species and algorithms, with AUC values ranging from 0.81 to 0.89, TSS values from 0.51 to 0.75, and Boyce index values from 0.80 to 0.97 (Table S3). For *L. multimaculatus*, the current model indicated high environmental suitability strictly restricted to coastal dune systems. However, projections for 2050 under both the SSP2-4.5 (intermediate) and SSP5-8.5 (pessimistic) scenarios predicted a near-total contraction of climatically suitable areas across its entire geographical range (Figure 4). These results suggest a critical sensitivity of *L. multimaculatus* to projected rising temperatures and altered precipitation patterns. Results for *L. wiegmannii* revealed a broad contemporary climatic niche, spanning the coastal region and the Argentine Arid Diagonal, which extends northwest–southeast (from 22°15′ S, 67°10′ W to 49°20′ S, 67°40′ W) from the Puna highlands to the Atlantic coast of Patagonia across the Southern Cone of South America. Projections for 2050 under both SSP scenarios predicted a significant redistribution of climatic suitability characterized by a marked geographic shift. Specifically, the models anticipate a loss of suitability along the Pampean and Uruguayan sandy coastlines, partially offset by an expansion toward the central-southern Monte shrublands and the western edge of the Arid Diagonal. Within the Eastern Barrier Dunes specifically, a generalized decline in suitable climatic conditions is projected relative to the baseline, with favorable areas persisting only in the northern sector. A paradoxical pattern emerged in this subregion: the pessimistic scenario (SSP5-8.5) retained higher levels of localized suitability compared to the intermediate scenario (SSP2-4.5), although both represent a significant net decrease relative to current baseline conditions (Figure 5).

## 4. Discussion

The findings of this study provide compelling evidence that the endemic habitat specialist *Liolaemus multimaculatus* and the widespread generalist *Liolaemus wiegmannii* respond differentially to projected changes in land-use/land-cover (LULC) patterns and climate. Our quantitative analysis highlights the severity of this response for the specialist, revealing that nearly 90% of the active dune habitat loss by 2050 occurs within areas already experiencing critical climatic niche contraction. This divergence aligns with regional assessments indicating that ecological specialization in Pampean dune lizards acts as a critical mediator of species sensitivity to structural landscape changes and climatic seasonality [43]. Specifically, our results comprehensively support our first hypothesis, demonstrating that LULC changes and habitat loss negatively impact the specialist *L. multimaculatus* to a greater extent than the generalist *L. wiegmannii*. This confirmed our first prediction, given that active dunes—the obligate habitat for the specialist—underwent a 20% historical retraction and are projected to decrease by an additional 17% by 2050. Our analysis confirmed that while afforestation was historically a dominant driver of active dune loss (14%), urban expansion is projected to become the primary force of anthropization by 2050, accounting for 25.97% of the regional territory. These results are consistent with previous findings on historical and projected LULC changes for the southern sector of Villa Gesell County and the northern sector of Mar Chiquita County [37]. The marked habitat loss in the Eastern Dune Barrier validates the role of urbanization and exotic afforestation as primary drivers of native habitat degradation, as documented by Austrich et al. [35], Block et al. [34], and Block et al. [42], who reported reduction and fragmentation of the native dune cover used by *L. multimaculatus* and *L. wiegmannii*. In particular, urban development, infrastructure construction, and artificial dune fixation have long been recognized as the major anthropogenic LULC drivers affecting dune systems globally, disrupting sedimentary equilibrium and causing coastal erosion and habitat fragmentation, among other impacts [104]. These anthropogenic pressures contribute to “coastal squeeze”, where ecosystems are trapped between expanding human infrastructure and rising sea levels, a phenomenon recently quantified globally [105], and which specifically restricts the landward migration necessary for temperate dune systems to maintain their ecological connectivity. These processes and their associated alterations to beach–dune dynamics and sediment budgets have been documented for the study area [36,37,49]. A large share of these LULC dynamics is amplified by tourism activity; approximately 50% of global tourism takes place in coastal destinations, generating an income of USD 3 trillion in 2023 [106]. Large tourist complexes, dedicated resort villages, and even coastal tourism-oriented cities are often built by flattening dune fields, removing native vegetation, and stabilizing dune sediments through the introduction of exotic species [107].

Regarding the abundance modelling component, we must explicitly acknowledge that the low pseudo-R^2^ values obtained for our abundance models (0.04 for *L. multimaculatus* and 0.01 for *L. wiegmannii*) represent a recognized limitation, reflecting the high stochasticity of field-based surveys and the exclusion of fine-scale environmental variables (e.g., microhabitat structure, substrate temperature) that were precluded by the requirements of our 2050 climate and land-use change projections [108]. While these values indicate that the evaluated landscape metrics explain only a modest fraction of the total variance, we argue that the results remain ecologically meaningful, as recent literature confirms that spatial abundance models often recover interpretable biological gradients despite low statistical power [109]. Our findings align with previous research on herpetofauna and stream fish, where similarly modest R^2^ values yielded management-relevant spatial patterns [110,111]. For *L. multimaculatus*, the identified spatial gradients are supported by independent ecological niche models, suggesting the capture of a genuine ecological signal, whereas the near-null fit for *L. wiegmannii* serves as an informative result indicating that the assessed landscape metrics are not the primary determinants of abundance for this habitat-generalist species [112]. Given the often-triangular nature of abundance–suitability relationships, our spatial outputs effectively capture relative ecological gradients rather than exact local counts, a conclusion supported by diagnostic confirmation that our Zero-Inflated Poisson structures correctly captured the data-generating process without significant bias [112]. Nevertheless, we acknowledge that these low explanatory values constrain the transferability of our predictions beyond the sampled domain, highlighting the need for future efforts to incorporate finer spatial grains and explicit detectability modeling to enhance local predictive accuracy [109].

Moreover, our second prediction was explicitly supported by abundance models showing a severe contraction of *L. multimaculatus* core areas driven by urban sprawl and exotic afforestation, while *L. wiegmannii* abundances remained relatively stable across the landscape. This finding strengthens the consensus that *L. multimaculatus* abundance depends strictly on active dune availability and is hindered by habitat loss across multiple scales [42,43]. While the abundance of *L. multimaculatus* exhibits high sensitivity to landscape fragmentation, *L. wiegmannii* demonstrates greater resilience to changes in landscape structure. However, the abundance and population dynamics of both lizard species were strongly influenced by the covariation of ambient temperature and relative humidity [61]. The combined effect of these environmental drivers on native reptiles is consistent with the “double threat” framework, where physiological sensitivity, low vagility, and restricted geographic ranges accelerate population collapse within anthropogenic matrices [27,28,29]. This aligns with broader biogeographic projections, where reptile populations globally are increasingly threatened by climate-driven shifts in habitat suitability [24], with many specialist species showing limited adaptive capacity compared to generalists, echoing the “erosion of diversity” reported in other lizard assemblages exposed to altered thermal niches [25]. The strong negative association of specialist reptile species with LULC change, resulting in compositionally altered landscapes, emphasizes that these adjacent land covers—especially exotic afforestation—function as a dispersal barrier, strongly confining the specialist to its core suitable habitat [113,114], thereby highlighting the critical importance of natural habitat structure at multiple scales [115]. The combined effect of these global change drivers is consistent with observations in other terrestrial vertebrates, where the combination of alterations in climate and landscape exacerbates extinction risk [8,9].

Despite *L. wiegmannii* associating with semi-fixed dunes, and projections for 2050 indicating a 23% loss of this native cover, the projected abundance of this generalist lizard remains stable. This pattern matches evidence that its abundance can associate positively with landscape heterogeneity promoted by a certain degree of fragmentation [43]. Generalists often present greater tolerance for disturbance and the ability to exploit structurally complex, heterogeneous landscapes [116]. The species’ inherent flexibility allows it to capitalize on complex patch edges, which likely facilitate dispersal and resource exploitation between nearby patches, a form of landscape complementation [117]. This finding aligns with observations of generalist herpetofauna benefiting from more geometrically complex patches [118], suggesting that, for this species, edge habitats and the surrounding matrix are utilized rather than acting as a strong barrier [119]. Conversely, structural degradation and loss of natural LULC—as documented globally—is a severe factor limiting the persistence of range-restricted species that cannot easily track suitable climatic niches [11]. In this sense, generalists often persist in human-modified environments, potentially leading to faunal homogenization [17,23].

Regarding climate change, the evidence fully supports our second hypothesis and confirms our third prediction, as ecological niche models projected a near-total contraction of climatically suitable areas for *L. multimaculatus* across its entire range in Atlantic coastal dune barriers of the Eastern Pampas and Northern Patagonia. This critical sensitivity to rising temperatures and altered precipitation patterns threatens to shift the niches of Pampean reptiles toward areas already under heavy land-use pressure, in alignment with reports by Di Pietro et al. [40]. Spatiotemporal variation in ecophysiology and life cycle can bias the predictions of correlative niche models by introducing biological responses that static models—typically based on correlations between presence and climate—fail to capture. For instance, *L. multimaculatus* populations may acclimatize their thermal preference to cope with geographic changes in the thermal environment, while adjusting their thermal physiology to cope with local climatic variations [120,121]. In addition, *L. multimaculatus* populations show a delay in sexual maturity and a decreased reproductive time, proportion of adult life, and net reproductive rate over the last 80–90 years, likely related to the increase in environmental temperatures [122]. If this trend continues, it could affect life history traits, becoming a serious threat for some populations, particularly those under anthropogenic pressure. Thus, vulnerability to global warming will not be uniform in space and time. It is important to consider that *L. multimaculatus* is intimately dependent on the specific habitat structure of active dunes that provide thermally suitable patches for thermoregulation [38] when interpreting the projections of our correlative niche models and land-cover change models. The limited dispersal capacity and physical landscape barriers inherent to range-restricted habitat specialists preclude effective niche tracking, as the degradation of specific land-cover types eliminates the potential for “jump dispersal” to emerging climatically suitable refugia [123]. Furthermore, global climate change is altering atmospheric processes, surface wind regimes, sea surface temperatures, and global ocean wave climates, among other drivers [124]. The spatial and temporal heterogeneity of these impacts causes beach–dune systems to be chronically and episodically remodeled across multiple scales [125]. This will directly influence dune-system dynamics, dune morphology, and the structure of associated vegetation communities [126].

Integrating correlative niche models with LULC change projections effectively bridges the gap between broad climatic patterns and structural landscape constraints to determine species survival, as synergistic effects of climate and land-use change drive broad-scale populational trends [9,19,20]. However, to further refine global change predictions, future studies should employ mechanistic frameworks that incorporate thermal and metabolic constraints [127,128]. Such mechanistic integration helps resolve the complex interplay between physiological limits and the structural loss of land cover, which can trap populations and prevent them from tracking shifting climatic envelopes. In this study, the simultaneous application of both modeling frameworks addresses the long-term trajectory of these species toward 2050 while utilizing their operational complementarity. Local abundance modeling (30 m pixel resolution, GLMs) quantifies proximate land-cover threats but remains geographically constrained, whereas regional niche modeling (1 km resolution) captures distal climatic shifts across the full species range but lacks local resolution. This methodological divergence directly reflects the multi-scale nature of global change processes rather than a structural limitation. Consequently, the convergence of both analytical signals toward an acute habitat deterioration for *L. multimaculatus* by 2050 strongly validates our overarching ecological diagnosis.

Regarding the methodological implementation, it is essential to clarify that the abundance modeling and niche modeling frameworks were not designed for statistical integration into a single combined model, as they constitute independent lines of evidence differing fundamentally in response variables (local abundance counts versus occurrence records), spatial scales (30 m versus 1 km resolution), predictor types (land-cover metrics versus bioclimatic variables), and modeling approaches (manually fitted GLMs versus automated SDM algorithms). Given these disparities, a joint statistical analysis would be methodologically inappropriate; instead, we employ a converging-lines-of-evidence approach, where each framework independently informs distinct dimensions of species vulnerability—proximate land-cover threats versus distal climatic shifts—and the directional agreement between these signals is interpreted as a robust pattern supported by independent evidence. Furthermore, our 2050 LULC projection represents a single deterministic trajectory derived from historical transition rates (1994–2022) using a Markov chain and Artificial Neural Network approach (MOLUSCE), which precludes the parameterization of alternative socioeconomic scenarios and means that uncertainty regarding future socioeconomic volatility is not formally propagated into our vulnerability estimates. This constraint is particularly relevant given that future coastal urbanization, while specified in local planning instruments, remains highly dependent on socioeconomic conditions and is intrinsically linked to artificial dune-fixation practices involving exotic species, a dynamic consistent with the findings of Garzo et al. [37], who documented how alternating phases of dune afforestation and subsequent deforestation for urban development mirror major macroeconomic cycles in Argentina. While these factors introduce substantial uncertainty, the current modeling remains grounded in robust environmental and regulatory criteria, representing a plausible projection of 2050 conditions. Ultimately, acknowledging these predictive limitations—including the need for future research to prioritize the integration of finer spatial grains, explicit detectability modeling, spatially explicit structures, and scenario-based LULC frameworks—is essential for a realistic interpretation of the conservation status of range-restricted specialists when integrating multiple global change drivers.

In the case of *L. wiegmannii*, our findings show a broader contemporary climatic niche, and it is projected that this niche will be displaced from the Eastern dune barrier inland toward the Arid Diagonal extending from the northwest–southeast of the Southern Cone of Argentina. This projection is in alignment with Sweeney and Jarzyna [23], who mentioned that species with broader niches possess greater resilience to global change. Nevertheless, Stellatelli et al. [39] documented that even the generalist *L. wiegmannii* may face physiological costs from LULC changes; the loss of thermal quality in modified habitats can lead to declines in the body condition of adults. In this sense, Dajil et al. [44] suggested that the ecological requirements of *L. wiegmannii* are not uniform across all life stages; juveniles are particularly sensitive to fine-scale habitat structure and resource availability, revealing hidden vulnerabilities in a species often assumed to be tolerant.

However, we identified potential caveats in the reliance on traditional species occurrence models to predict the impacts of climate change on lizard species. A primary limitation of these models is their tendency to assume ecological uniformity across a species’ range and life cycle, often overlooking the sublethal physiological costs incurred by individuals in modified landscapes. While species distribution models categorize *L. wiegmannii* as a resilient generalist based on broad distribution patterns, they fail to account for the loss of thermal quality at the microscale. Furthermore, the ontogenetic shift in habitat requirements identified in recent research by Dajil et al. [44] suggests that occurrence-based frameworks may inadvertently mask the high sensitivity of juveniles to fine-scale structural degradation. By ignoring these life-stage-specific vulnerabilities and the energetic trade-offs associated with LULC change, traditional modeling approaches likely overestimate the capacity for “niche tracking” and underestimate the long-term extinction risk for populations currently persisting under suboptimal thermal and structural conditions [12].

The integration of LULC and climate drivers confirms our fourth prediction by identifying “high-risk” zones where low climatic suitability converges with intense landscape transformation [9]. For *L. multimaculatus*, the restriction of high environmental suitability to a specific LULC class solely within coastal systems makes the projected climatic contraction a precursor to potential regional extinction. In this sense, the reduction in high-abundance areas to remnant sectors, even within protected areas like the Faro Querandí Reserve, warns of an extreme vulnerability scenario. Our projections are supported by recent findings by Dajil et al. [61], who report that habitat fragmentation and climatic seasonality operate differentially; the specialist suffers critical loss where active dunes are replaced by anthropogenic matrices, while the generalist exploits small-scale heterogeneity. As sea level rises, the rigidity of urbanization or artificially fixed dune fields prevents the inland migration of coastal systems, creating a “coastal squeeze” exacerbated by global change [129,130]. This regional manifestation of “coastal squeeze” is consistent with the global analysis by Lansu et al. [105], confirming that infrastructure development significantly reduces the space available for natural shoreline retreat, thereby accelerating the loss of sensitive dune habitats. In the long term, these systems tend to lose their capacity for sediment exchange, increasing their hydrometeorological vulnerability, coastal erosion, and habitat loss [105]. This dynamic reinforces the need to prioritize the conservation of remaining active dune patches as both structural and climatic refugia to ensure habitat connectivity [42]. Our findings underscore that ecological specialization is the primary determinant of differential sensitivity to the biophysical stressors of the Anthropocene [16]. Therefore, given the advancement of urban development goals, it is imperative to implement management strategies that account for the synergies between climate and LULC spatial distribution to prevent the collapse of endemic specialists [20,29]. Effective protection must expand beyond current boundaries, ensuring that areas of high future suitability are not irreversibly transformed before species can occupy them [46]. Failure to address these interactions will likely result in faunal homogenization dominated by a few resilient species at the expense of specialized endemic lineages [17,29].

## 5. Conclusions

In summary, this study demonstrates that ecological specialization is the primary determinant of vulnerability to global change within the Pampean Eastern Dune Barrier, as evidenced by the contrasting trajectories of the endemic specialist *Liolaemus multimaculatus* and the generalist *Liolaemus wiegmannii*. By integrating fine-scale abundance modeling (30 m pixel resolution) to quantify local land-use and land-cover impacts with broad-scale ecological niche modeling (~1 km^2^ resolution) to capture regional bioclimatic shifts, we provide a robust, multi-scalar diagnostic of species persistence toward 2050. While the low pseudo-R^2^ values in our abundance models indicate that we capture only a fraction of the variation in local count data, our diagnostics confirm the robustness of the model structure, and the results provide ecologically coherent and management-relevant insights into spatial abundance gradients. We acknowledge that the low explanatory power of these models limits their transferability beyond the current sampled domain, highlighting a clear need for future research to incorporate finer-scale variables and explicit detectability metrics. Importantly, these spatial outputs must be interpreted as relative ecological gradients rather than absolute abundance predictions, reflecting the inherent stochasticity of field-based surveys. The convergence of these independent methodological frameworks—addressing both proximal habitat loss from urban expansion and distal climatic niche contraction—reinforces our central hypothesis. However, to move beyond these correlative signals and fully account for the “coastal squeeze,” future research must incorporate mechanistic niche modeling to explicitly link physiological constraints, such as operative temperatures and hydric balance, to the rapidly shifting biophysical properties of the dune habitat. Our findings confirm that while *L. wiegmannii* maintains resilience through landscape heterogeneity, *L. multimaculatus* faces a synergistic “double threat” that signals a looming faunal homogenization and potential extinction debt. These results underscore an urgent need for integrated conservation strategies that prioritize the restoration of active dune remnants and the implementation of local and provincial legislation to restrict invasive woody species and dune fixation initiatives based on the deliberate introduction of rapid-growth exotic species, ensuring that management frameworks account for the complex interplay between physiological limits and anthropogenic landscape transformation to prevent the collapse of range-restricted endemic lineages.

## Figures and Tables

**Table 1:** Land-use and land-cover (LULC) changes in the Eastern Barrier Dunes of the Pampas region, Argentina. Data are presented in hectares (ha) and relative percentage (%) for the years 1994, 2022, and projected 2050. The delta symbol (Δ) denotes the percentage point difference between the final and initial years for the periods 1994–2022 and 2022–2050. References: Active Dunes: sparsely vegetated upper beach, active foredunes, and active inland dunes; Semi-Fixed Dunes: continuous natural grassland, clumped herbs, and shrubs; Interdune Depressions: vegetation associated with seasonal or permanent flooding; Afforested Dunes: areas dominated by exotic tree species; Urban Areas: built-up environments and human settlements; Beaches: coastal sandy interfaces; Water Bodies: internal aquatic systems; Roads: transport infrastructure.

	1994	2022	2050	Δ 1994–2022	Δ 2022–2050
LULC	ha (%)	ha (%)	ha (%)	%	%
Urban Areas	5627.88 (12.18)	7191.27 (15.56)	12,000.06 (25.97)	+3.38	+10.41
Forested dunes	2286.72 (4.95)	4470.93 (9.68)	4052.52 (8.77)	+4.73	−0.90
Active Dunes	10,431.00 (22.58)	8308.35 (17.98)	6905.79 (14.95)	−4.59	−3.04
Semi-Fixed Dunes	7350.66 (15.91)	6612.12 (14.31)	5113.80 (11.07)	−1.60	−3.24
Interdune Depressions	8214.48 (17.78)	7749.18 (16.77)	6277.05 (13.58)	−1.01	−3.19
Water bodies	10,339.11 (22.38)	10,638.36 (23.02)	10,636.74 (23.02)	+0.65	−0.004
Beaches	1807.92 (3.91)	1090.44 (2.36)	1086.75 (2.35)	−1.55	−0.008
Roads	144.90 (0.31)	120.78 (0.26)	108.72 (0.24)	−0.05	−0.03

## Data Availability

The data supporting the findings of this study are openly available in Zenodo at https://doi.org/10.5281/zenodo.21348143 under a Creative Commons Attribution 4.0 International (CC BY 4.0) license.

## Supplementary Materials

The following supporting information can be downloaded at https://www.mdpi.com/article/10.3390/biology15141152/s1, Figure S1: Geographic distribution of occurrence records for *Liolaemus multimaculatus*. Figure S2: Geographic distribution of occurrence records for *Liolaemus wiegmannii*. Figure S3: Residual diagnostics for the *Liolaemus multimaculatus* model generated using the DHARMa R package. Figure S4: Residual diagnostics for the *Liolaemus wiegmannii* model generated using the DHARMa R package. Table S1: Pearson correlation coefficients for explanatory variables. Table S2: Pearson correlation coefficients for bioclimatic variables. Table S3: Predictive performance metrics for the ecological niche models. Table S4: Sensitivity analysis of binarization threshold methods for the Random Forest (RAF) suitability models of *Liolaemus multimaculatus* and *L. wiegmannii*.
