# Supplementary material for: Vulnerability of Pampean Coastal Lizards to Global Change: Divergent Responses of Endemic Specialists and Widespread Generalists
Source: Biology (Basel). 2026 Jul 15;15(14):1152. doi: 10.3390/biology15141152 (PMC13405765; doi:10.3390/biology15141152)
Supplement: Supplementary file 1 [file biology-15-01152-s001.zip › biology-4387388-supplementary.pdf]

Supplementary Materials

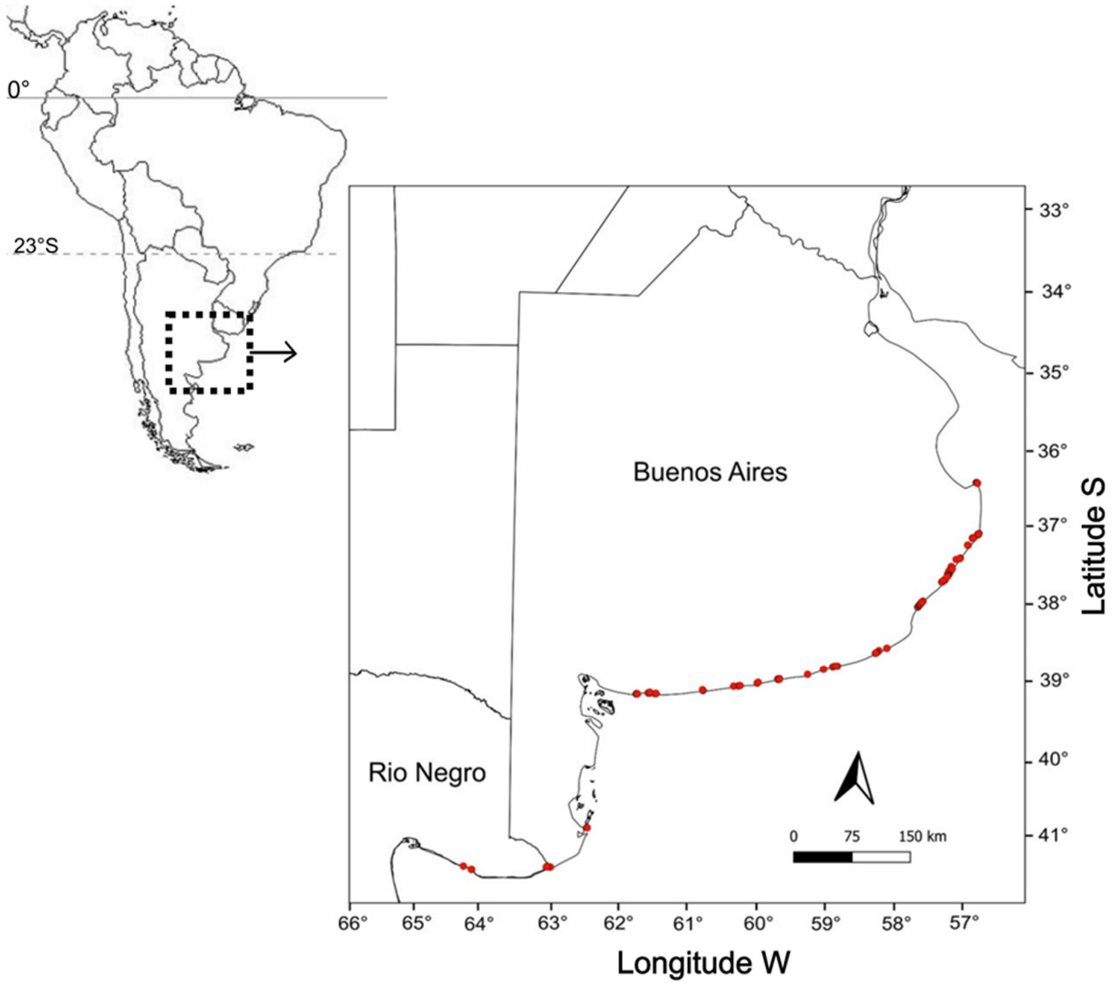

Figure S1. Geographic distribution of occurrence records for Liolaemus multimaculatus. The map displays occurrence localities (n = 282) across the species' known range in the coastal dunes of Buenos Aires and Río Negro provinces, Argentina. Red circles indicate specific occurrence localities compiled from Cei [76], Vega and Bellagamba [77,78], Etheridge [79], Stellatelli et al. [80], Abdala et al. [81], and Block et al. [42]. The inset map illustrates the study area relative to the South American continent.

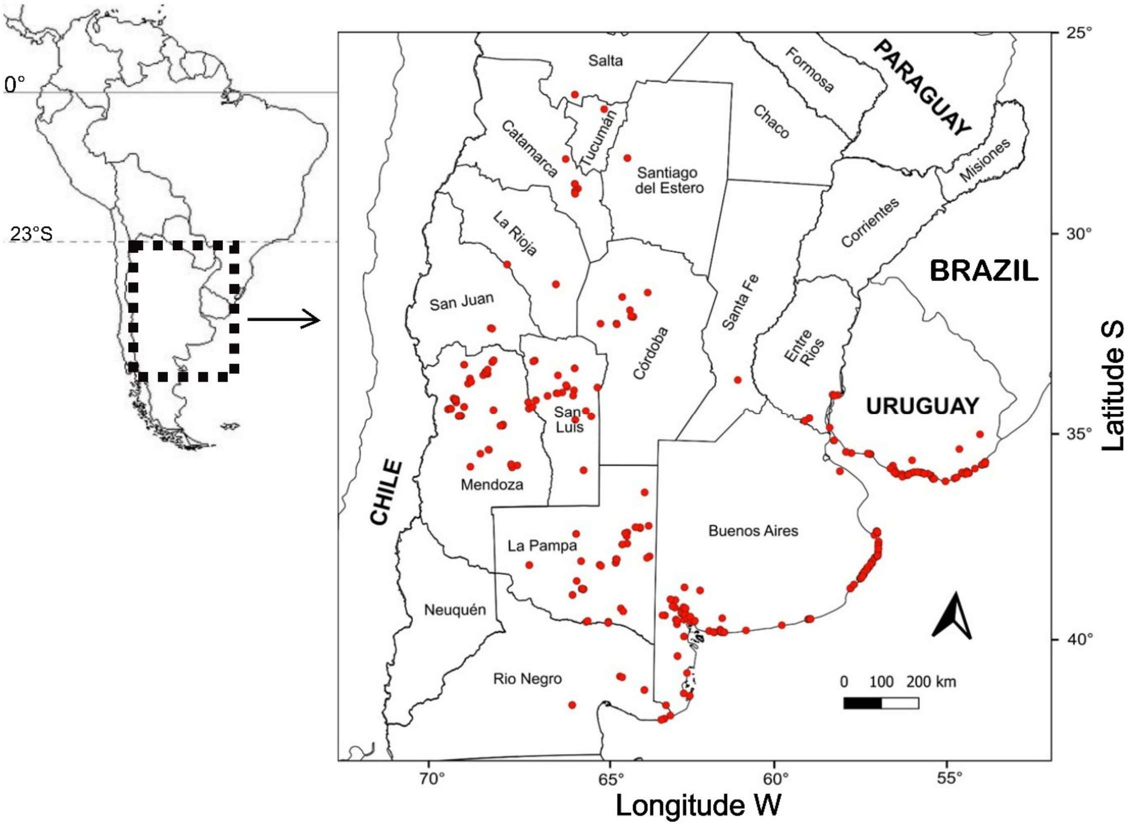

Figure S2. Geographic distribution of occurrence records for Liolaemus wiegmannii. The map displays occurrence localities (n = 783) throughout the distribution range in Argentina and Uruguay. Red circles denote occurrence/presence localities compiled from Cei [76], Achaval and Olmos [82], Vega and Bellagamba [77], Martori et al. [83], Etheridge [79], Scrocchi et al. [84], Stellatelli et al. [38,39], Verrastro et al. [85], Villamil et al. [86], Abdala et al. [81], and Williams et al. [87]. The inset map highlights the study region within South America; provincial

and national boundaries are labeled for reference.

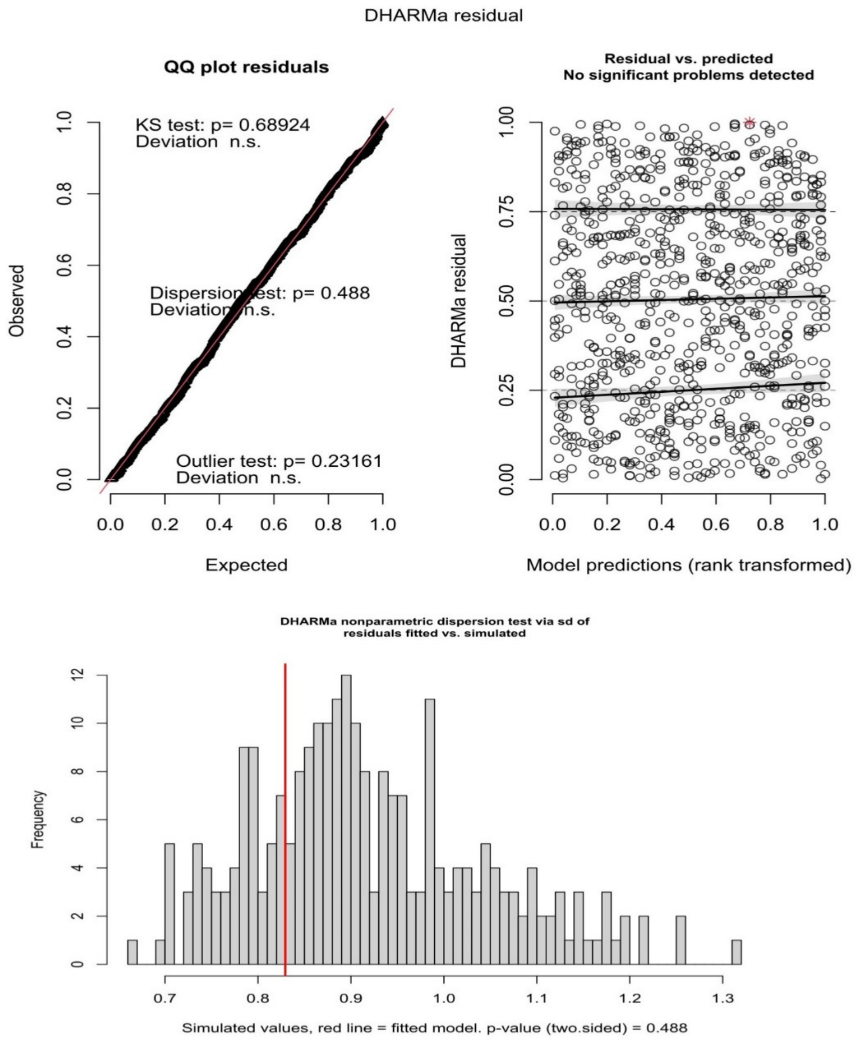

Figure S3. Residual diagnostics for the *Liolaemus multimaculatus model*, generated using the DHARMa R package. The figure displays three diagnostic plots to assess model fit. The left panel shows the quantile-quantile (QQ) plot of simulated residuals; the lack of significant deviations from the 1:1 line indicates that the model residuals follow the expected distribution (K-S test: p = 0.69; Outlier test: p = 0.23). The middle panel displays simulated residuals against predicted values (rank-transformed), showing a uniform distribution with no visible patterns, suggesting that the model adequately captures the data structure without systematic bias. The right panel presents the dispersion test, comparing the observed standard deviation

of residuals against those from simulated datasets; the result confirms no evidence of over- or under-dispersion (p = 0.48). Together, these diagnostics support a robust model fit and adherence to underlying distributional assumptions.

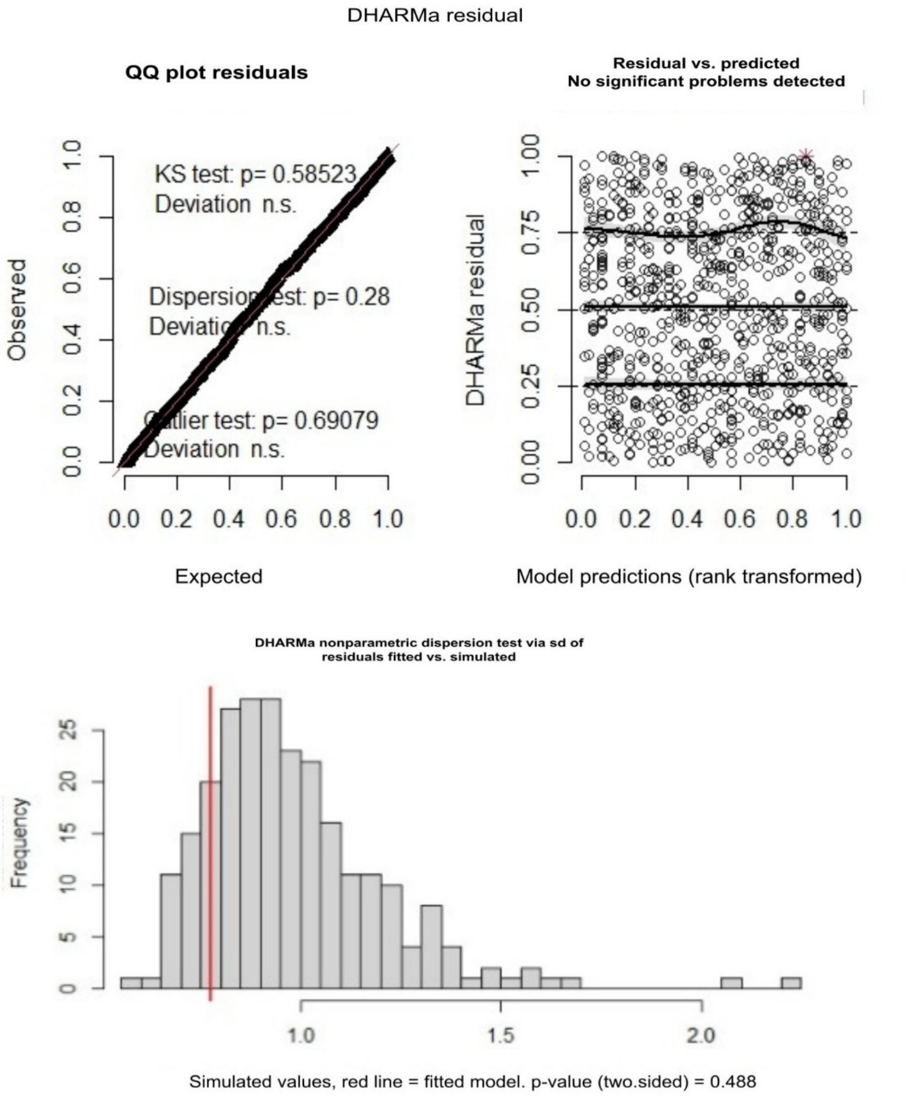

Figure S4. Residual diagnostics for the *Liolaemus wiegmannii* model, generated using the DHARMa R package. The figure displays three diagnostic plots to assess model fit. The left panel shows the quantile-quantile (QQ) plot of simulated residuals; the lack of significant deviations from the 1:1 line indicates that the model residuals follow the expected distribution (K-S test: p = 0.59; Outlier test: p = 0.69). The middle panel displays simulated residuals

against predicted values (rank-transformed), showing a uniform distribution with no visible patterns, suggesting that the model adequately captures the data structure without systematic bias. The right panel presents the dispersion test, comparing the observed standard deviation of residuals against those from simulated datasets; the result confirms no evidence of over- or under-dispersion ($p = 0.28$). Together, these diagnostics support a robust model fit and adherence to underlying distributional assumptions.

Table S1. Pearson correlation coefficients for explanatory variables. Values (*r*) represent pairs of predictors analyzed across the four zones of the eastern dune barrier of the Pampas. Biogeographic and topographic variables include: DEM, Digital Elevation Model; Slope, the steepness or incline of the terrain surface; Aspect, the compass direction that the terrain slope faces; Dist. Urb, Euclidean distance to urban settlements; and NDVI, Normalized Difference Vegetation Index for the year 2022.

	Zone 1	Zone 2	Zone 3	Zone 4
DEM vs Slope	0.36	0.35	0.27	0.47
Aspect	0.03	-0.02	0.01	0.07

Dist. Urb	-0.15	-0.15	-0.30	0.01
NDVI	0.19	-0.05	0.51	0.12
Slope vs				
Aspect	0.02	-0.04	0.04	0.05
Dist. Urb	0.42	0.29	0.45	0.51
NDVI	-0.02	-0.19	0.14	-0.01
Aspect vs				
Dist. Urb	-0.01	-0.04	-0.01	0.01
NDVI	0.08	0.16	0.06	0.02
Dist. Urb vs NDVI	-0.16	-0.30	-0.44	0.24

Table S2. Pearson correlation coefficients for bioclimatic variables. Values (*r*) calculated for bioclimatic factors within the calibration areas of (a) *Liolaemus multimaculatus* and (b) *Liolaemus wiegmannii*. Parameters derived from WorldClim include: BIO1, Annual Mean Temperature; BIO4, Temperature Seasonality; BIO6, Minimum Temperature of Coldest Month; BIO7, Temperature Annual Range; BIO12, Annual Precipitation; BIO15, Precipitation Seasonality; and BIO18, Precipitation of Warmest Quarter.

a- L. multimaculatus

Bioclimatic Variables	BIO1	BIO6	BIO7	BIO12
BIO1	-	0.03	0.13	-0.34
BIO6	-	-	-0.54	0.41
BIO7	-	-	-	-0.39
BIO12	-	-	-	-

b- L. wiegmannii

Bioclimatic Variables	BIO1	BIO4	BIO12	BIO15	BIO18
BIO1	-	-0.02	0.46	0.26	0.29
BIO4	-	-	-0.33	-0.12	-0.30

BIO12	-	-	-	-0.58	0.59
BIO15	-	-	-	-	0.41
BIO18	-	-	-	-	-

Table S3. Predictive performance metrics for the ecological niche models. Evaluation of *Liolaemus multimaculatus* and *Liolaemus wiegmannii* models implemented via Random Forest (RAF), Maximum Entropy (MaxEnt), and Generalized Linear Models (GLM) using the flexsdm R package. Performance was evaluated using a 5-fold cross-validation (80/20% split). Evaluation metrics include Area Under the Curve (AUC), True Skill Statistic (TSS), Jaccard Index, Boyce Index, and Inverse Mean Absolute Error (IMAE). Values in bold indicate the best-performing algorithm for each species.

Species	Algorithm	AUC	TSS	JACCARD	BOYCE	IMAE
L. multimaculatus	Random Forest	0.85	0.73	**0.73**	0.88	0.68
	MaxEnt	**0.89**	**0.75**	0.72	**0.97**	0.68
	GLM	0.88	0.72	**0.73**	0.95	**0.71**
L. wiegmannii	Random Forest	**0.86**	**0.64**	**0.58**	0.80	**0.76**
	MaxEnt	0.85	0.60	0.54	**0.86**	0.70
	GLM	0.81	0.51	0.49	**0.86**	0.70

Table S4. Sensitivity analysis of binarization threshold methods for the Random Forest (RAF) suitability models of *Liolaemus multimaculatus* and *Liolaemus wiegmannii*. Values represent means ± standard deviation across five spatial cross-validation partitions (part_sblock, flexsdm R package v1.4.0; Velazco et al., 2022). Threshold methods include: max_sens_spec, the threshold that maximizes the sum of sensitivity and specificity; max_jaccard, the threshold that maximizes the Jaccard index; max_sorensen, the threshold that maximizes the Sorensen index; equal_sens_spec, the threshold that equates sensitivity and specificity; and lpt, the lowest presence threshold. The method used to generate binary suitability maps and area estimates in the main text is indicated in bold.

Species	Threshold method	Mean threshold ± SD	TSS	Sorensen	Jaccard
L. multimaculatus	max_sens_spec	0.318 ± 0.243	0.778	0.855	0.759
L. multimaculatus	max_jaccard	0.304 ± 0.261	0.768	0.873	0.787
L. multimaculatus	max_sorensen	0.304 ± 0.261	0.768	0.873	0.787
L. multimaculatus	equal_sens_spec	0.171 ± 0.159	0.686	0.824	0.716
L. multimaculatus	lpt	0.158 ± 0.168	0.633	0.841	0.749
L. wiegmannii	max_sens_spec	0.151 ± 0.077	0.563	0.69	0.538
L. wiegmannii	max_jaccard	0.191 ± 0.143	0.559	0.696	0.544
L. wiegmannii	max_sorensen	0.191 ± 0.143	0.559	0.696	0.544

L. wiegmannii	equal_sens_spec	0.113 ± 0.067	0.489	0.643	0.488
L. wiegmannii	lpt	0.006 ± 0.008	0.187	0.542	0.378
